# Early Recognition and Successful Treatment of Anti-synthetase Syndrome

**DOI:** 10.7759/cureus.21786

**Published:** 2022-01-31

**Authors:** Arabi Rasendrakumar, Aakanksha Khanna, Smita Bakhai

**Affiliations:** 1 Department of Internal Medicine, University at Buffalo Jacobs School of Medicine and Biomedical Sciences, Buffalo, USA; 2 Department of Rheumatology, Dartmouth-Hitchcock Medical Center, Lebanon, USA

**Keywords:** inflammatory myopathy, polyarthritis, proximal muscle weakness, anti-jo-1 antibodies, polymyositis, anti-synthetase syndrome, interstital lung disease

## Abstract

Anti-synthetase syndrome is an autoimmune disorder that is characterized by inflammatory myopathy, non-erosive polyarthritis, interstitial lung disease in addition to the presence of anti-aminoacyl t-RNA synthetase antibody. It can have variable presentations posing a major diagnostic challenge. Recognition of this syndrome is crucial for appropriate, timely therapy to prevent morbidity and mortality. We report the case of a 55-year-old male who initially presented to the emergency department (ED) with sudden onset shortness of breath, low-grade fever, dry cough, fatigue, and severe arthralgia. He was diagnosed with community-acquired pneumonia and was discharged with antibiotics. He then presented to his primary care physician (PCP) with worsening symptoms. A computed tomography (CT) scan of the chest showed the presence of patchy bilateral airspace opacities and infiltrates. He had elevated inflammatory markers and anti-nuclear antibodies (ANAs). Pulmonary function test (PFT) showed a restrictive pattern with a reduction in lung volumes. Further workup revealed the presence of anti-Jo-1 antibodies. In addition, a muscle biopsy was obtained which showed inflammatory myopathy. Lung biopsy was consistent with interstitial fibrosis. The diagnosis of the anti-synthetase syndrome was made and the patient was promptly started on high-dose prednisone and cyclophosphamide which was later switched to azathioprine and tacrolimus due to resistance and side effects. The patient’s symptoms improved significantly with the current treatment without any other complications. This case highlights the importance of a thorough history and physical exam by PCP. Prompt communication and care coordination between PCP and specialists (rheumatologist and pulmonologist) are essential to expedite diagnostic testing and initiate treatment early in this disorder.

## Introduction

Anti-synthetase syndrome is a rare autoimmune disorder that is characterized by the presence of antibodies directed against an aminoacyl transfer RNA synthetase [1] . Proximal muscle weakness is typically the first manifestation. The diagnosis is characterized by combination of the following clinical features, in varying severity: inflammatory myopathy (including dermatomyositis [DM] or polymyositis [PM]), interstitial lung disease (ILD) [2] , polyarthritis, fever, Raynaud’s phenomenon, and/or hyperkeratotic fingertips (mechanic’s hand). In many cases, it is associated with elevated muscle enzymes and abnormal muscle biopsy showing immune myopathy with perimysial pathology with or without fascicular atrophy.

Six anti-aminoacetyl transfer RNA (t-RNA) synthetase autoantibodies have been identified: anti-histidyl-tRNA synthetase (anti-Jo-1), anti-threonyl-tRNA (anti-PL7), anti-alanyl-tRNA (anti-PL12), anti-isoleucyl-tRNA (anti-OJ), anti-glycyl-tRNA (anti-EJ) and anti-asparaginyl-tRNA (anti-KS) with anti-synthetase syndrome [1,3-4]. These autoantibodies are useful markers for the diagnosis and prediction of symptoms. The most common and well recognized of which is anti-Jo-1 (anti histidyl t-RNA synthetase) found in 20%-30% of such patients [2,4]. The infrequency with which it is encountered makes anti-synthetase syndrome a formidable diagnostic challenge.

## Case presentation

**Figure 1:**
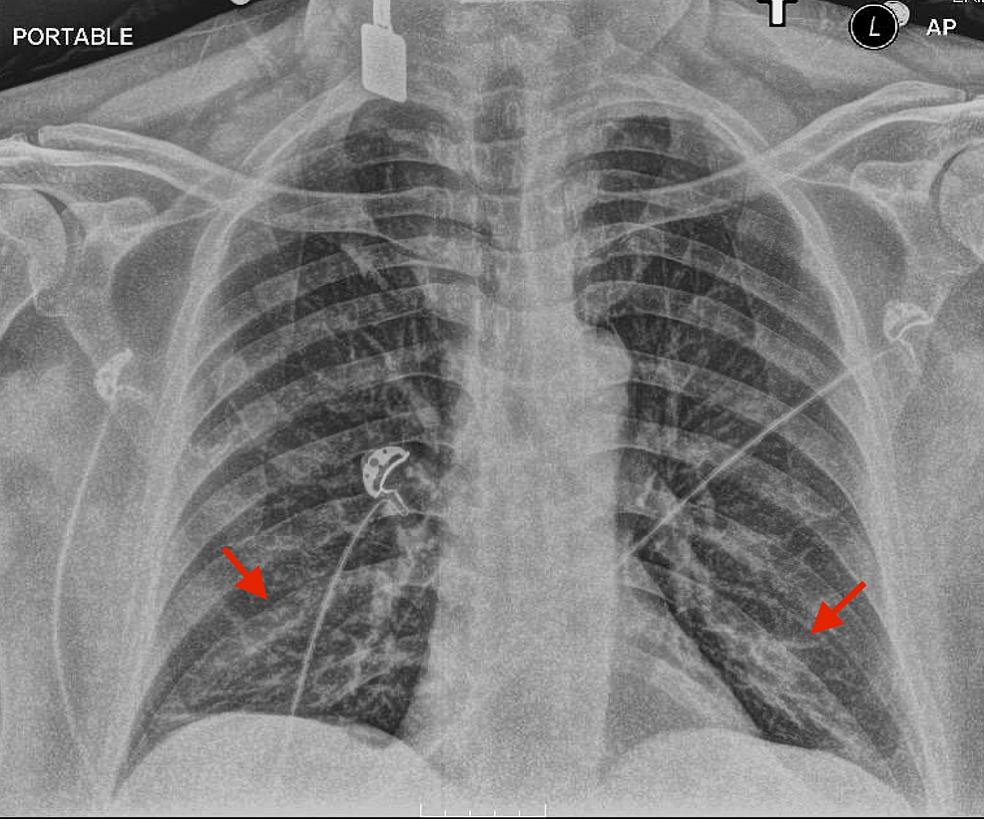
Initial chest x-ray showing interstitial and airspace opacities in the bilateral lower lung fields (red arrows).

A 55-year-old male presented to the emergency department (ED) due to sudden onset of shortness of breath, fever, chills, dry cough, fatigue, and severe arthralgia. Initial chest x-ray showed bilateral lower lung field infiltrates (Figure 1). The patient was diagnosed with community-acquired pneumonia and was discharged with a course of oral antibiotics.

Three days after he was evaluated at the ED, he went to his primary care physician (PCP) due to worsening dyspnea, fever, malaise, and severe arthralgia in spite of taking the antibiotic as prescribed. He reported severe pain involving his bilateral knees, ankles, and hands. He denied chest pain, palpitations, weight loss, nausea, vomiting, diarrhea, constipation, numbness, tingling, vision change, photosensitivity, morning stiffness, rash, oral or genital lesions.

Past medical history was significant for hypertension and depression. No significant past surgical history or family history. He had a 20-pack year smoking history but quit two years ago. No alcohol or recreational drug use. He worked at a home supply store, checking the inventory of supplies, and did not have any occupational exposure to dust, silica, asbestosis, or chemical irritants. 

On physical examination, the patient was in acute distress. He appeared ill, fatigued, and short of breath at rest. He was hypoxic with oxygen saturation at 86% on room air which improved to 99% after he was placed on 2 L of oxygen. Respiratory examination revealed crackles at the lung bases bilaterally. A musculoskeletal exam revealed proximal muscle weakness over the deltoid, biceps, and quadriceps muscles with nonspecific tenderness of his bilateral knee, wrists, and elbows. Polyarticular synovitis was detected in bilateral wrists and knee joints; there were no associated features of rash, lower extremity edema, cyanosis, or clubbing.

The PCP was extremely concerned by the worsening presentation of the patient in view of progressive dyspnea, recurrent fevers, and arthritis. The PCP considered infectious causes (including drug-resistant pneumonia, pneumocystis pneumonia, and tuberculosis), ILD, pulmonary involvement from a possible connective tissue disorder, sarcoidosis, and possible underlying malignancy. A wide array of laboratory tests (as detailed in Table 1), a repeat chest x-ray, and computed tomography (CT) of the chest were ordered. The patient was sent home with 2 L of oxygen by nasal cannula with the instruction to go to the ED if symptoms worsen.

**Table 1:** Hematological and biochemical parameters of the patient obtained after the initial primary care physician visit.

Parameter	Result	Reference Range
White blood cell count (WBC)	11.5 k/cumm	4.8-10.8
Neutrophils	33.6%	40.0-75.2
Lymphocytes	54%	16.0-51.0
Hemoglobin	14.3 g/dL	14.0-18.0
Red blood cell count (RBC)	4.78 M/cumm	4.7-6.1
Platelets	293 k/cumm	130-400
Serum sodium	140 mmol/L	
Serum potassium	4.2 mmol/L	
Serum chloride	107 mmol/L	96-108
Serum bicarbonate	23 mmol/L	19-30
Serum calcium	8.7 mg/dL	8.4-10.2
Serum urea	21 mg/dL	6-20
Serum creatinine	1.1 mg/dL	0.7-1.2
Total protein	7.2 g/dL	6.6-8.7
Serum albumin	4.1 g/dL	3.4-4.8
Serum total bilirubin	0.5 mg /dL	<1.1
Alanine aminotransferase (ALT)	18 Units/L	<42
Aspartate aminotransferase (AST)	15 Units/L	<38
Alkaline phosphatase (ALP)	47 Units/L	40-129
Uric acid	4.7 mg/dL	3.4-7.0
Serum lactate dehydrogenase (LDH)	398 Units/L	135-225
Erythrocyte sedimentation rate (ESR)	12 mm/hr	0-15
C- reactive protein (CRP)	10.9 mg/dL	0.1-0.5
Anti-nuclear antibodies titre (ANA)	1:360	<1:40
Thyroid stimulating hormone (TSH)	0.74 µIU/mL	0.27-4.20
Total creatine kinase (CK)	1026 Units/L	38-174
Rheumatoid factor (RF)	< 10 IU/mL	01-15
Aldose	7.7 Units/L	1.2-7.6
Angiotensin converting enzyme (ACE)	24 Units/L	8-53
Human immunodeficiency virus (HIV) 1 and 2 antibody	Nonreactive	
Hepatitis B surface antibody	Negative	
Hepatitis C antibody	Negative	

**Figure 2:**
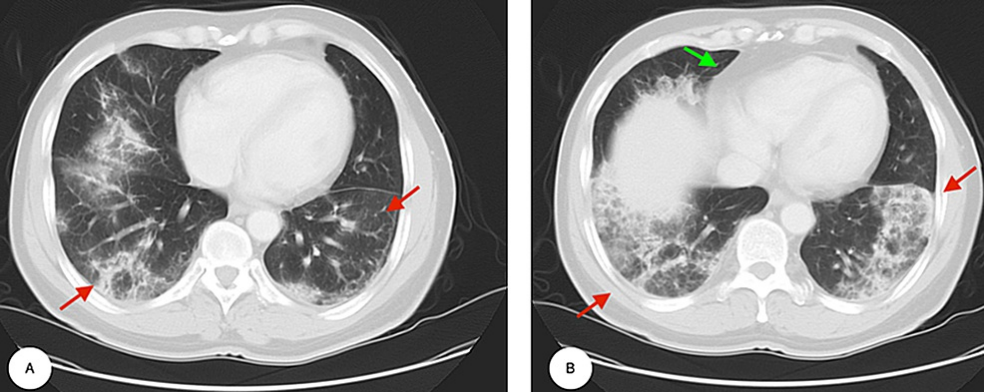
Multi-slice computed tomography (CT) of the chest. (A) Horizontal tomography slice of the mid thorax and (B) lower thorax demonstrating interlobular reticular septal thickening and ground-glass opacity in bilateral mid and lower lung lobes (red arrows) with mediastinal lymphadenopathy (green arrow).

A review of the first set of investigations showed elevated C-reactive protein (CRP), creatine kinase (CK), anti-nuclear antibody (ANA), and lactate dehydrogenase (LDH). Repeat chest x-ray showed bilateral infiltrates which was worse compared to the one done in the ED. CT chest obtained one week after his initial visit showed patchy bilateral airspace opacities and interstitial infiltrates (Figures 2A, 2B).

In suspicion of possible acute onset ILD with simultaneous presentation of inflammatory myopathy, referral to a local rheumatologist was placed. The patient was seen in person by the rheumatologist within three weeks after the initial PCP visit. During this encounter, the patient had worsening dyspnea, proximal muscle weakness, arthritis, and Raynaud’s syndrome. The patient was still requiring supplemental oxygen at this time. On physical examination, the patient was unable to arise more than five times from the chair without the use of his arms. Muscle strength was 4/5 in the deltoids, biceps, triceps, and quadriceps muscles bilaterally. Synovitis was noted on the wrists, elbows, and left second & third metacarpophalangeal (MCP) joints. He did not have Gottron papule over his hands or heliotrope rash. Comprehensive autoimmune serology panel including anti-double-stranded DNA antibody (anti-ds DNA), anti-smith, anti-ribonucleoprotein (anti-RNP), anti-histidyl-tRNA synthetase (Anti-Jo 1), anti-threonyl-tRNA (anti-PL7), anti-alanyl-tRNA (anti-PL12) antibodies were obtained. It was all negative except for the anti-Jo-1 antibody which was elevated at 584 Units (normal level <1.0 Units). 

In view of progressive symmetrical proximal muscle weakness, ILD, elevated CK (1026 ), and positive anti-Jo-1 antibody (584); the diagnosis of the anti-synthetase syndrome was suspected.

**Figure 3:**
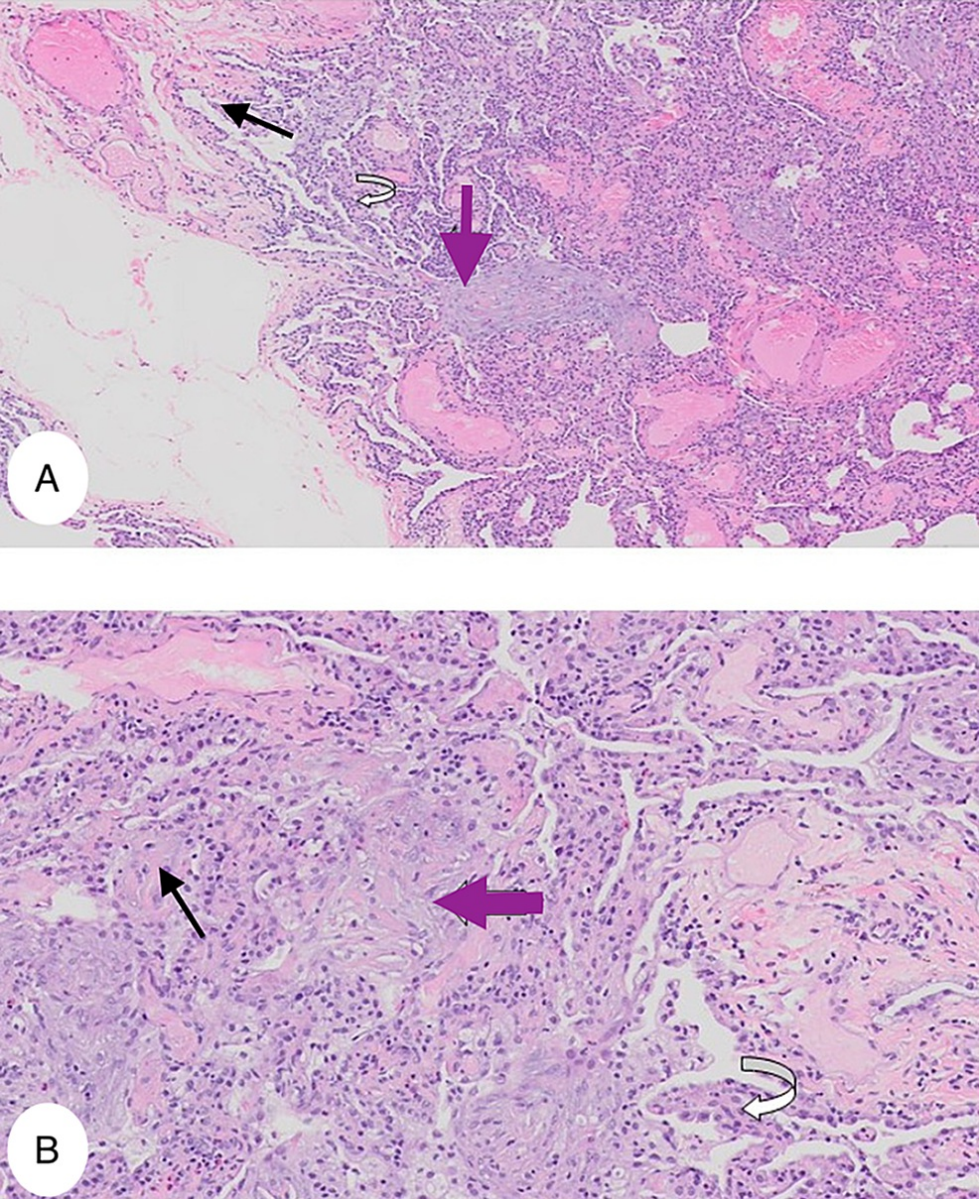
Histopathology from a trans-bronchial sample of the right lower lobe lung. Both slides (A, B) illustrate the lung parenchyma with granulation tissue suggestive of interstitial pulmonary fibrosis. (A) is a low power view and (B) is a high power view of the specimen showing intra-alveolar (black arrow) and interstitial fibrosis (purple arrow) and alveolar epithelial hyperplasia (white curved arrow).

He was referred to a pulmonologist to assess his lung function. His initial pulmonology function test (PFT) showed a restrictive pattern with a reduction in lung volumes. Diffusing capacity for carbon monoxide (DLCO), forced expiratory volume (FEV1) and forced vital capacity (FVC) were all reduced and were consistent with ILD. He subsequently underwent bronchoscopy with trans-bronchial lung biopsy of the right lower lobe, which showed localized fibrosis mainly in the intra-alveolar space suggestive of interstitial pulmonary fibrosis (Figures 3A, 3B).

He subsequently underwent a muscle biopsy of his left calf to check for inflammatory myopathy. The pathology reports detailed necrotic and regenerating fibers together with small foci of lymphocyte inflammation. It showed endomysial inflammation with CD 8+ cells with necrotic muscle fiber consistent with PM. 

**Figure 4:**
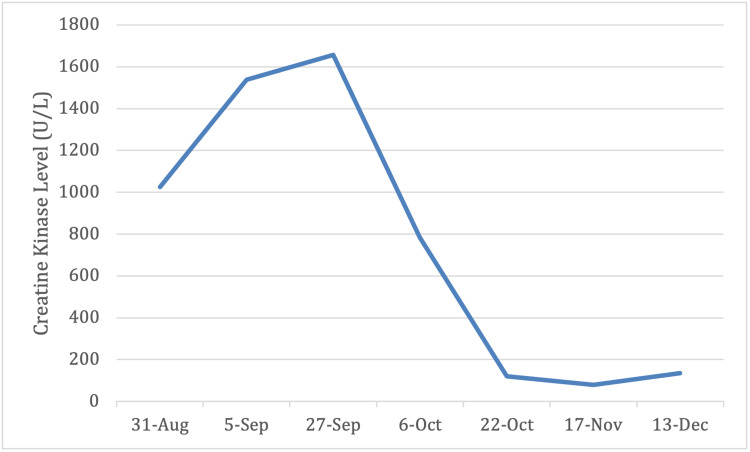
Trend of creatine kinase (CK) through diagnostic and treatment period (within six-month period).

The patient was officially diagnosed with anti-synthetase syndrome based on Solomon et al [5] diagnostic criteria. He had the presence of anti-Jo-1 antibody along with ILD and PM, which are part of the major criteria. Interstitial lung disease was supported by the patient's CT chest and PFT results while PM was confirmed with a muscle biopsy as detailed above. Patient was subsequently started on high dose prednisone (1mg/kg/day) 80mg daily and cyclophosphamide (1-2mg/kg/day) 80mg daily. He responded significantly well with improvement in his muscle strength and respiratory status as his oxygen saturation improved to 95% on room air; no longer requiring supplemental oxygen. The patient’s CK level paralleled disease activity (Figure 4). It peaked at 1,657 within one month of symptom onset and trended down to 80 after one month on cyclophosphamide and prednisone therapy.

**Figure 5:**
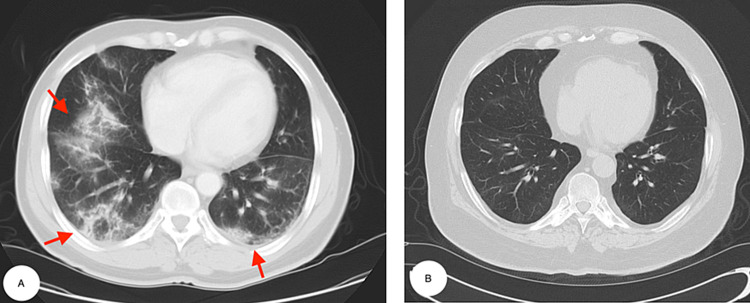
CT scan of the chest before treatment and after two years on treatment. (A) Patchy bilateral ground-glass opacities and infiltrates (red arrows). (B) Resolution of the infiltrates.

The patient had significantly improved PFT within four months of diagnosis. Repeat CT scan of the chest after two years showed significant improvement and complete resolution of ILD (Figures 5A, 5B).

During his treatment, he developed hematuria as a result of cyclophosphamide therapy, which was stopped and was switched to tacrolimus 1mg twice daily. He also developed complications of chronic steroid use including diabetes, osteoporosis, and worsening of his hypertension. In view of this, prednisone was slowly tapered to a low dose. Azathioprine 80mg daily was added for additional immunosuppression. For his steroid-induced diabetes, he was treated with metformin and insulin. For his osteoporosis, he was started on alendronate once weekly. He remained stable on low-dose prednisone, azathioprine, and tacrolimus. He was in remission without any further complications under the care of rheumatology and PCP for six years before he relocated to another state.

## Discussion

Anti-synthetase syndrome should be suspected in cases of unexplained ILD with signs of inflammatory myositis (Table 2) [1]. It can have variable presentations posing a major diagnostic challenge for physicians. Recognition of this syndrome is critical for appropriate, timely therapy to prevent morbidity and mortality from ILD [4,6].

**Table 2:** Diagnostic criteria of anti-synthetase syndrome.

	Connors et al. (2010) [7]	Solomon et al. (2011) [5]
Required criteria	Presence of anti-aminoacyl tRNA synthetase antibody	Presence of anti-aminoacyl tRNA synthetase antibody
Additional criteria	One or more of the following: Raynaud’s phenomenon, arthritis, interstitial lung disease, fever and/ or mechanic’s hands	2 major criteria or 1 major plus 2 minor criteria. Major criteria: Interstitial lung disease, polymyositis/ dermatomyositis. Minor criteria: Raynaud’s phenomenon, arthritis, mechanic’s hands

In this case, there were some diagnostic errors that contributed to a delay in the patient’s diagnosis when he initially presented to the ED (Table 3). This was later realized by the PCP when he presented after his ED visit. His PCP avoided diagnostic errors such as anchoring heuristic bias by not adhering to the initial diagnosis of pneumonia and looked at the patient’s entire clinical presentation to appropriately diagnose and initiate treatment.

**Table 3:** Diagnostic errors that may have contributed to delay in diagnosis by the ED physician.

Bias/ Diagnostic errors	Definition	Example from case
Availability heuristic	Judging by ease of recalling past cases	Clinical diagnosis of pneumonia at initial presentation with fever, dyspnea, tachypnea, and abnormal chest X-ray
Information Avoidance	Ignoring relevant information and useful clues for timely diagnosis	Failure to recognize the patient’s entire clinical presentation in which he had significant arthralgia and muscle weakness preceding his respiratory symptoms

ILD is not only a frequent manifestation but also a significant prognostic factor in these patients. Prevalence of ILD has been estimated to occur in 65% of patients with inflammatory myopathies [8]. High-resolution CT chest and PFT s are sensitive tools to detect early ILD [9]. Recognition is critical to prevent mobility and mortality from ILD [10]. This patient presented simultaneously with PM and ILD. He responded well initially with high dose prednisone and cyclophosphamide; however, was later switched to low dose prednisone, azathioprine, and tacrolimus due to developing side effects/ adverse effects. Patients suffering from anti-synthetase syndrome usually require multimodal immunosuppression therapy. It primarily involves high-dose corticosteroid along with either azathioprine, mycophenolate mofetil, tacrolimus, and/or rituximab [1,11].

Misdiagnosis of this condition could delay treatment and result in worsening of the patient’s condition. As compared to a case report where a 60-year-old woman who had PM for 20 years with remitting fever, arthritis, and muscle weakness. Her diagnosis of the anti-synthetase syndrome was delayed and had to be on a prolonged course of treatment to manage her symptoms and disease activity with multiple trials of immunosuppressive agents [12]. 

In 2013, a retrospective study of 202 patients with anti-synthetase syndrome found that 10-year survival was 70% for Jo-1 antibody-positive patients versus 49% for non-Jo-1 patients [6]. In addition, the most common cause of death was due to pulmonary fibrosis and pulmonary hypertension. About 6% had to undergo lung transplantation. It is also interesting to note that in a 2015 survival analysis study, out of 45 patients with the anti-synthetase syndrome; 14% died five years after the diagnosis was made [6]. Patients that died had significantly decreased FVC and DLCO. This illustrates the importance of early recognition and prompt treatment to prevent the progression of ILD. High-resolution CT chest and PFT are sensitive tools to detect early ILD.

It is also crucial to monitor for disease activities and complications related to therapy. Serial CK, periodic imaging, and PFT help monitor the patient’s symptoms and guide therapy which was illustrated by this case. Patients with anti-synthetase syndrome require close follow-up to monitor symptoms, adverse effects, and complications as a result of the disease process itself or due to immunosuppressive treatment.

## Conclusions

This case illustrates the importance of early diagnosis of the anti-synthetase syndrome and prompt treatment which results in early remission of the disease process. It also highlights the importance of a thorough history and physical exam by the PCP and prompt communication/collaboration between specialists (PCP, rheumatologist, and pulmonologist) to expedite diagnostic testing and treatment.
